# Revealing the Importance of Iron Aerogel Features as Electrocatalysts for the Oxygen Reduction Reaction

**DOI:** 10.3390/gels11030154

**Published:** 2025-02-20

**Authors:** Judith González-Lavín, Ana Arenillas, Natalia Rey-Raap

**Affiliations:** Instituto de Ciencia y Tecnología del Carbono, INCAR-CSIC, 33011 Oviedo, Spain; judith.g.lavin@incar.csic.es

**Keywords:** iron aerogel, electrocatalyst, oxygen reduction reaction, transition metal, carbon functionalization

## Abstract

Metal nanoparticles supported in carbon materials are the traditional electrocatalyst currently used in many applications. However, these composite materials have many problems associated with the optimization of both components for the specific application, besides the stability of the mixture. Self-supported metallic materials may be an interesting strategy in order to avoid the traditional carbon supports; however, these metallic materials should present highly active surface area. Iron aerogels are presented in this work as effective and affordable unsupported electrocatalysts. The combination of their metallic structure with high porosity (i.e., 85 m^2^ g^−1^ and 0.45 cm^3^ g^−1^ of mesopore volume), due to their interconnected tridimensional structure, leads to a great activity versus the oxygen reduction reaction. A method for producing iron aerogels based on microwave-assisted sol–gel methodology is presented. The incorporation of carbon functionalities to the iron aerogels seems to clearly influence the mechanism of the reaction, favoring the direct mechanism of the oxygen reduction reaction and thus notably improving the performance of the electrocatalysts. Chemical vapor deposition seems to be an adequate methodology for incorporating carbon functionalities to the transition metal structure without affecting the tridimensional network and leading to current densities over 4 mA cm^−2^ and great stability even after 10,000 s.

## 1. Introduction

Metal nanoparticles (MNPs) supported in carbon materials have been employed as electrocatalysts for many decades due to their unique properties and versatility such as large surface area, selectivity, and electrocatalytic activity [1,2,3]. However, despite being widely used, these electrocatalysts have some important drawbacks and challenges that should be addressed to improve their electrochemical performance. Generally, carbon supports may have a well-developed porous structure, ensuring the proper diffusion of reactants and products from and to the active sites. MNPs provide the active sites, which is their key role in the catalytic reaction, but they present limited accessibility to the active sites due to porosity restriction [3]. The combination of both materials (i.e., carbon support and MNPs) is therefore the most common solution. However, electrocatalysts should have a good interaction between MNPs and the carbon supports to avoid metal leaching. This usually arises from the propensity of MNPs to detach from the carbon support matrix under harsh conditions of operation, during which carbon supports can suffer from oxidation [4,5]. This phenomenon not only compromises the stability of the electrocatalysts but also results in the loss of electrocatalytic activity, diminishing the efficiency of the material over time. In addition, the properties of the carbon surface may affect the dispersion and stability of the MNPs and, hence, their long-term performance. The preparation of well-dispersed MNPs on carbon supports is essential to achieve a uniform distribution and prevent their passivation, aggregation, or structural changes which may contribute to the gradual attenuation of their electrochemical performance [6]. To address these issues, several strategies have been studied such as varying the amount of the metal salt precursor, using additives or surfactants, or anchoring isolated metal atoms on the support [6,7]. Moreover, alternative materials to MNPs supported into carbon materials have been developed such as metal oxides [8], metal nitrides, sulfides, or phosphides, metal–organic frameworks [9], or bulk metals [10,11], which have been demonstrated to exhibit high electroactivity toward several electrochemical reactions [12,13]. However, these materials may require high activation energy and complex and costly synthesis processes [14]. They also may present low stability under harsh operation conditions (degradation issues) and low specific surface area [15,16], hindering diffusion processes. Therefore, researchers continue to explore novel electrocatalysts that may address all these challenges to enhance their electrochemical performance.

A novel strategy to solve all these drawbacks focuses on the development of freestanding materials like metal aerogels (MAs) which offer significant advantages in electrocatalytic applications, mitigating some of the problems mentioned above for supported MNPs [17,18,19,20,21]. MAs are highly porous materials with a unique nanostructured network, characterized by low density and high surface area. Thus, they may provide abundant active sites for electrochemical reactions, as the contact between the reactants and the electrocatalyst is maximized [21]. In addition, MAs can be engineered to have designed pore size distributions and porosity, promoting diffusion-controlled reactions and facilitating the efficient mass transfer of reactants and products within the electrocatalytic system. These features minimize diffusion limitations and improve reaction kinetics, leading to enhanced performance and selectivity [17,22,23,24,25]. On the other hand, MAs exhibit excellent thermal stability and remarkable mechanical strength and resilience [26,27,28,29], retaining their structural integrity and electrocatalytic activity even at high temperatures. Thus, they are able to maintain their durability under dynamic operating conditions, reducing the risk of structural deformation or active site loss [30]. Based on these advantages, several MAs have been developed in recent decades, especially noble metal aerogels (NMAs) due to their tremendous potential for electrochemical applications [22,31,32]. However, NMAs present certain drawbacks associated with the cost and scarcity of noble metals, as well as their market dependence due to geographical location limitations [32]. Therefore, the partial or total replacement by transition metals is emerging as a promising strategy to maximize the performance-to-cost ratio.

Most synthesis routes to produce transition metal aerogels (TMAs) involve complex processes such as solvent exchange or long-lasting washing steps, and the use of toxic chemicals such as epoxides, that complicate or even limit their scale-up and commercial success. Iron has been widely studied as an electrocatalyst due to its abundance, low cost, and electroactivity in oxygen reduction reactions (ORRs). However, most of the materials reported in the literature involve complex procedures and the use of toxic components or high-value materials. Thus, Fe-octaethylporphyrin requires a Au support [33], and the options proposed exhibited very low current densities [33,34]. The combination with carbon nanotubes (CNTs) has also been suggested [35], and high current densities are achieved in this case. However, a long process is needed, and the use of toxic reactants such as N,N-dimethylformamide is required. To address this issue, the microwave-assisted synthesis of iron aerogels (FeAs) has been recently proposed as a simple process that allows researchers to control their morphological and textural properties [36]. Notwithstanding, there are still some critical points to achieve the rational design and control of all the properties as it is achieved with aerogels of different natures such as silica or carbon aerogels [37,38,39,40], allowing them to be used in a wide range of applications. Therefore, this work aims to demonstrate the potential of FeAs as electrocatalysts toward the ORR, one of the most important reactions in fuel cells and metal–air batteries. In the case of fuel cells, the need to find an effective electrocatalyst that avoids the use of Pt is mandatory to drive the massive use of these devices. On the other hand, the development of metal–air batteries is essential for developing the needed energy storage devices that are not based on the use of lithium [41].

In this work, FeAs were functionalized with carbon atoms for the first time by employing different techniques, resulting in materials with different surface functionalities. The electrochemical performance of these functionalized materials was compared with composites involving carbon and iron aerogels, and bulk metal iron, demonstrating that developing novel approaches for incorporating surface functionalities into transition metal aerogels is of paramount importance to enhance their applicability in electrochemical applications.

## 2. Results and Discussion

### 2.1. Physicochemical Characterization

**Figure 1:**
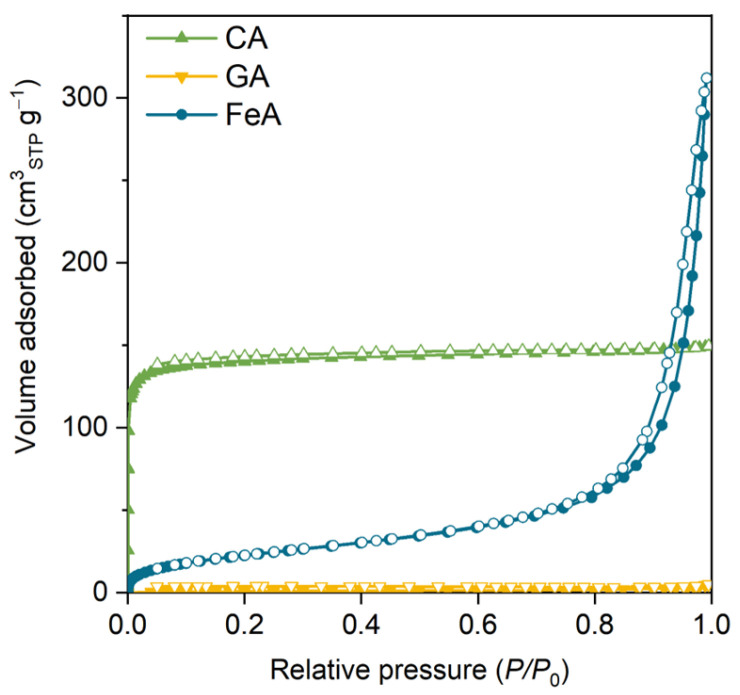
N_2_ adsorption–desorption isotherm of the carbon aerogel (CA), graphene aerogel (GA), and iron aerogel (FeA). Colored and empty marks correspond to the adsorption and desorption branches, respectively.

The N_2_ adsorption–desorption isotherms of the main three aerogels (carbon aerogel (CA), graphene aerogel (GA), and FeA) are shown in Figure 1. Sample CA exhibits a type I isotherm attributed to microporous materials. The high volume adsorbed at low relative pressures is indicative of a material with high specific surface area (565 m^2^ g^−1^) and volume of micropores (0.22 cm^3^ g^−1^). These values are similar to those commonly observed for carbon aerogels obtained from RF [42,43].

The volume adsorbed for GA is almost negligible, indicating that this sample does not have microporosity. GA is mainly composed of randomly connected graphene sheets recovered by carbon aerogel obtained from RF. However, the percentage of RF is quite low (8 wt.%), so there is not enough mass to form a microporous carbon gel [44]. In addition, CA and GA do not show a hysteresis loop at medium–high relative pressure, which suggests that these samples do not have small mesopores. Notwithstanding, they can present some large mesopores or macropores that cannot be measured by this technique (corroborated by SEM, as discussed below). The isotherm of FeA is a type II isotherm, attributed to micro–macroporous materials with a high specific surface area of 85 m^2^ g^−1^ in comparison to metal oxide materials [45,46]. This sample also shows a thin hysteresis loop at high relative pressures, indicating the presence of medium–large mesopores (around 30 nm). This feature is particularly important, as these mesopores are often referred to as feeder pores because they facilitate diffusion and access to the microporosity and thus to the active sites. In this sense, the external surface area (i.e., S_ext_ shown in Table S1) gives an idea of the accessible surface area. The presence of mesopores in FeA makes almost the entire S_BET_ easily accessible (S_BET_ ≈ S_ext_), whereas, in the case of CA, it is not. Based on these results, it can be inferred that CA, GA, and FeA are micro–macroporous, exclusively macroporous, and micro–meso–macroporous materials, respectively.

**Figure 2:**
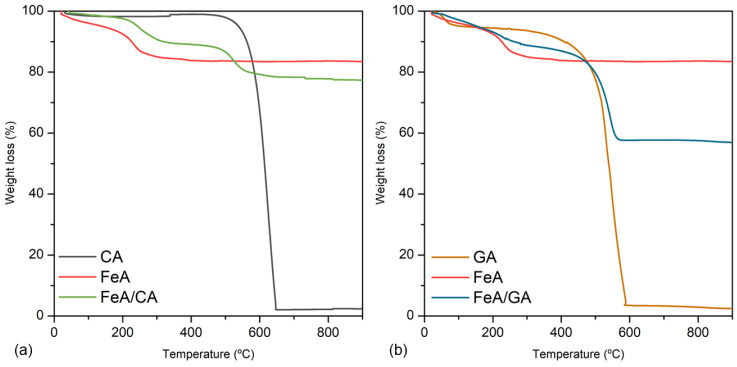
Thermogravimetric analysis measured in air for CA, FeA, and FeA/CA (**a**), and for GA, FeA, and FeA/GA (**b**).

The loading and stability of the composites involving these three materials (CA, GA, and FeA) were analyzed by TGA. Figure 2a,b show the mass loss in air of CA, FeA, and FeA/CA, and of GA, FeA, and FeA/GA, respectively. All TGA profiles follow their typical thermal decomposition processes in air atmosphere. CA is stable at temperatures below 460 °C, a temperature at which it starts to burn, being completely burned out at 650 °C. FeA exhibits a progressive weight loss up to 200 °C, which can be attributed to the removal of labile oxygen-containing functional groups adsorbed into the aerogel surface, exhibiting a high thermal stability at temperatures higher than 200 °C. The TGA curve of the FeA/CA composite is a combination of CA and FeA profiles, which has a weight loss of 10% at around 500 °C, consistent with the CA content employed to prepare the composite. On the other hand, the thermal decomposition pattern of GA presents some differences in comparison to CA, even though both samples are mainly composed of carbon. The weight loss of 5% observed at temperatures lower than 100 °C is assigned to the removal of physically adsorbed water. As the temperature increases, the material remains stable until 300 °C, a temperature at which starts to burn, being completely burned out at 590 °C. These temperatures are lower than those observed for CA, suggesting that the graphene aerogel is less thermally stable and more reactive.

The TGA profile of the composite prepared with this carbon material is a combination of GA and FeA curves. However, the total weight loss is about 30%, higher than the GA content employed to prepare the composite. This can be explained by the high reactivity of GA in air along with the exfoliation of the graphene sheets, which can contribute to a loss of mass due to vigorous exfoliation process. This can also be inferred from the TGA profile of GA, in which the weight loss is not a vertical line but occurs gradually between 300 and 600 °C, suggesting that, in the composite sample, the weight loss due to the vigorous exfoliation process is also dragging out part of the metallic aerogel structure, which takes place along with the burn-out of GA, achieving a weight loss higher than 10%.

**Figure 3:**
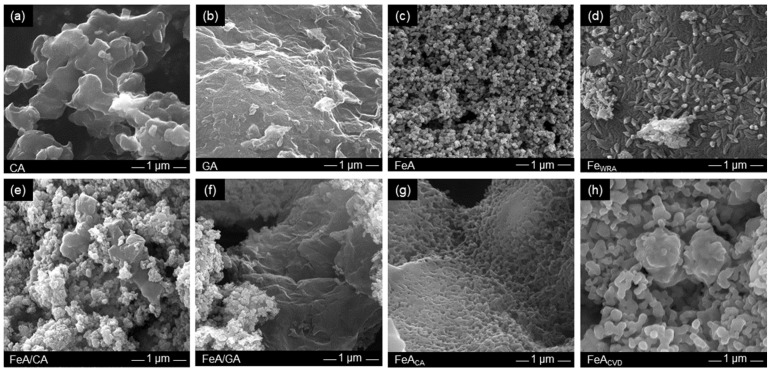
SEM images of the (**a**) carbon aerogel (CA), (**b**) graphene aerogel (GA), (**c**) iron aerogel (FeA), (**d**) iron compound reference Fe_WRA_, (**e**) composite FeA/CA, (**f**) composite FeA/GA, (**g**) FeA doped with citric acid (FeA_CA_), and (**h**) FeA doped with toluene by CVD (FeA_CVD_).

The morphology of these samples was observed by SEM (Figure 3). CA is formed by interconnected clusters of ca. 500 nm which leave large voids between them, i.e., macropores. This type of morphology is commonly observed for carbon aerogels obtained from the carbonization of organic RF aerogels [47]. In fact, the size of the clusters and hence the size of the pores can be designed by controlling the synthesis conditions [42,48]. In this work, these conditions were selected to obtain a macroporous material, as detailed in the Materials and Methods section, in order to compare it with the GA sample which is made up of randomly connected graphene sheets. The voids between these sheets are larger than those in CA, so they can only be observed at lower magnification (Figure S1).

Conversely, the morphology of FeA (Figure 3c) involves interconnected clusters that leave smaller voids between them, i.e., smaller macropores than those shown in CA and GA. This structure of interconnected nodules characteristic of aerogels differs from that observed for sample Fe_WRA_, which was not prepared via the sol–gel method. Its structure (Figure 3d) is compact, composed of small elongated formations of individual particles similar to rice grains. This demonstrates the importance of performing the sol–gel route to obtain a well-interconnected porous structure in aerogels to be employed as unsupported electrocatalysts. Regarding the composites (Figure 3e,f), a mixture of the morphology of the structures employed (FeA with CA or GA, respectively) can be observed. The functionalized iron aerogels have very different morphologies. In the case of FeA_CA_ (Figure 3g), the nodules appear to be more cross-linked than in the FeA sample, while in the CVD functionalization, they appear to grow by sintering in the form of more elongated clusters. These variations in morphology are probably due to the different temperatures employed in each functionalization process.

**Figure 4:**
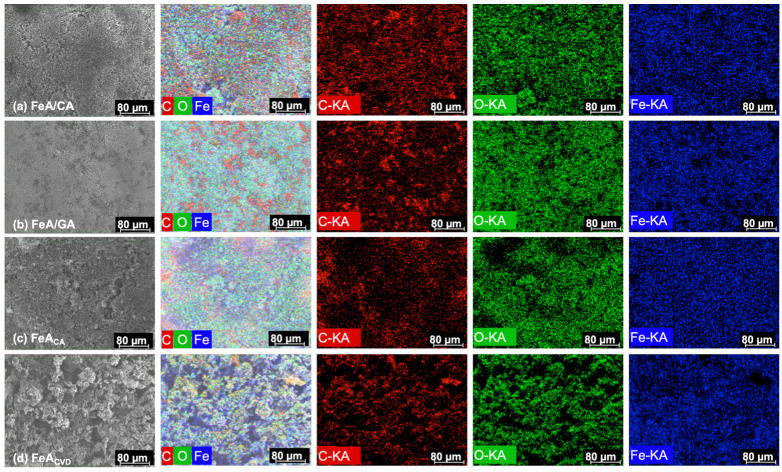
SEM images and energy-dispersive X-ray (EDX) mapping analysis of composite FeA/CA (**a**), composite FeA/GA (**b**), FeA doped with citric acid (**c**), and FeA doped with toluene by CVD (**d**).

SEM-EDX mapping, whose images are shown in Figure 4, was used to examine and map the main elements that made up the samples. The results confirmed the presence of well-dispersed carbon, oxygen, and iron atoms (K type series) in the proportions shown in Table 1. The proportion between the three elements is similar for both composites, while in the functionalized sample, the main element was iron. The functionalization by CVD resulted in a higher amount of carbon incorporated into the structure in comparison with the citric acid treatment, while the proportion of iron was quite similar. This could be due to the temperature employed during the CVD process (i.e., 700 °C), which may eliminate oxygen functionalities, leading to a decrease in oxygen content and the subsequent increase in carbon content in the final material.

### 2.2. Electrochemical Performance

**Figure 5:**
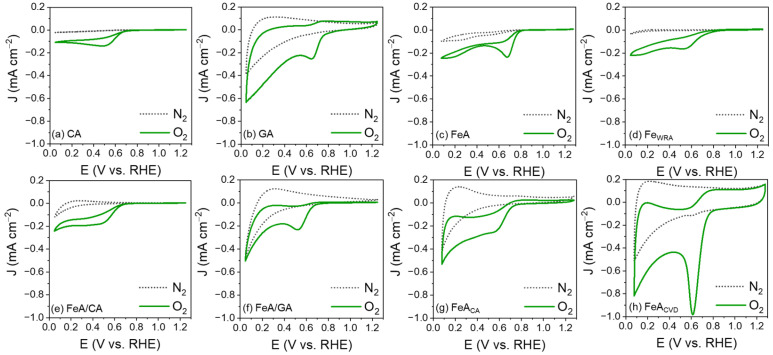
CVs measured in N_2_- and O_2_-saturated 0.1 M KOH electrolyte for the carbon aerogel (**a**), the graphene aerogel (**b**), the iron aerogel (**c**), the iron particles (**d**), the iron–carbon aerogel composite (**e**), the iron–graphene aerogel composite (**f**), the iron aerogel functionalized with citric acid (**g**), and the iron aerogel functionalized by chemical vapor deposition (**h**).

The electroactivity of the electrocatalysts was evaluated by cyclic voltammetry (CV) in a 0.1 M KOH electrolyte saturated in N_2_ and O_2_. Regardless of the material, none of the CVs measured in the N_2_-saturated electrolyte show any current peaks, while in the O_2_-saturated electrolyte, a defined peak appears, attributed to the reduction of oxygen. This peak appears with different current densities and potentials for each sample, indicating differences in their electroactivity. Sample CA exhibits the lowest electroactivity (Figure 5a), which is slightly enhanced by adding FeA into its structure (Figure 5e). However, its electroactivity remains uncompetitive and lower than that of FeA, demonstrating the potential of engineering self-supported metal structures. In the case of GA, a broadening of the voltammetry curve is observed compared to the carbon aerogel, accompanied by a higher capacitance (Figure 5b). This phenomenon arises because charge storage occurs through two mechanisms. On one hand, the double-layer capacitance, due to ion adsorption, is enhanced by the high porosity of the sample. On the other hand, pseudocapacitance, resulting from surface redox processes, is promoted by the oxygenated functional groups (-OH, -COOH, etc.) present in the GA sample. By forming the composite with the iron aerogel, the broadening is reduced, and the reduction peak becomes more defined, indicating that the main redox mechanism takes place at the active sites of the iron (Figure 5f). On the other hand, both FeA and Fe_WRA_ are electroactive toward the ORR, as iron is a catalytic material able to electrochemically react with oxygen, as long as iron atoms are accessible. Nonetheless, the performance of the iron aerogel (Figure 5c) surpasses that of Fe_WRA_ (Figure 5d). These results can be attributed to the combination of the morphology of the aerogel and its high porosity, which favors a fast mass transfer and increases the number of available active sites. In addition, the chemical composition of FeA also improves the electron transfer pathways and provides more reactive sites, leading to enhanced electrocatalytic activity, while the sample made of individual nanoparticles (Fe_WRA_) displays worse performance. The control in the synthesis process to design the local environment around the active sites leads to a defined morphology, demonstrating the importance of engineering transition metals into aerogel structures. In addition, the electroactivity toward the ORR of the unique structure of the aerogels can be further improved by functionalizing them with carbon atoms. The functionalization with citric acid results in a minimal improvement in the reduction peak current density (Figure 5g), while the CVD strategy highly improves the potential and the current density, leading to a high-performance electrocatalyst (Figure 5h). The current density of the reduction peak increases up to 1 mA cm^−2^, which is 4 times more intense than that in the undoped FeA sample. This may be due to the modification of the surface chemistry, as the anchoring of carbon atoms may have allowed for more efficient coordination for oxygen reduction, due to the bonding of the Fe d orbitals with the C π orbitals [49].

**Figure 6:**
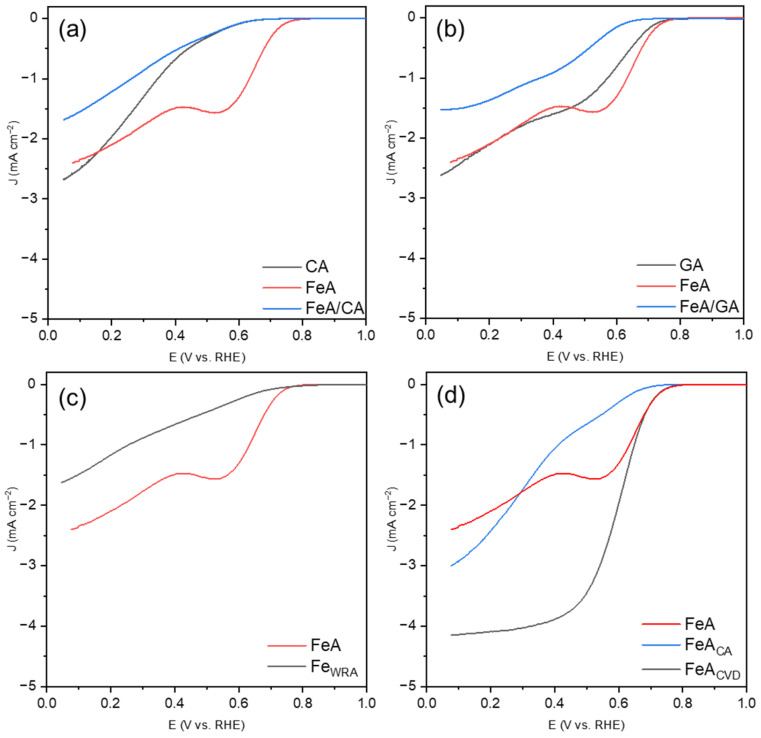
LSVs at 1600 rpm in KOH 0.1 M after subtracting the N_2_-saturated solution results from those obtained in an O_2_-saturated solution, measured with a scan rate of 5 mV s^−1^ for (**a**) CA, FeA, and the composite FeA/CA, (**b**) GA, FeA, and the composite FeA/GA, (**c**) iron samples FeA and Fe_WRA_, and (**d**) FeA and the doped samples FeA_CA_ and FeA_CVD_.

Linear sweep voltammetries (LSVs) for the samples studied are shown in Figure 6. The CA sample (Figure 6a) exhibits an LSV characteristic of a partial oxygen reduction route, from molecular oxygen to peroxide, without complete reduction to water. The reaction at the electrode is governed by slow kinetics, resulting in charge-transfer-controlled current, which prevents CA from reaching a diffusion-limited current regime. When the iron aerogel is added (which operates via a mechanism related to a 2 + 2 electron pathway and exhibits a notably enhanced onset potential), the LSV does not improve. This may be because the kinetic limitations of CA hinder the effect of FeA. In the case of the graphene aerogel (Figure 6b), the onset potential for GA is improved with respect to CA, but the mechanism is not yet the most favorable. The combination FeA/GA seems to show a plateau (indicating a one-step mechanism), although the current densities remain very low (i.e., far from the theoretical value of J = −6 mA·cm^−2^ for Pt/C). The study of the behavior of the iron-based materials separately, FeA and Fe_WRA_ (Figure 6c), allows researchers to distinguish the different electrocatalytic responses of these materials. The electrocatalytic response of Fe_WRA_ is very poor, showing a partial mechanism, which may be attributed to the low availability of active sites for catalyzing the reaction and a high energy barrier. However, the aerogel, with its porous structure, displays a well-defined two-step mechanism. Although the kinetics are slow, involving the partial breaking of the O=O bond, the aerogel provides more active sites compared to the Fe_WRA_ sample. Finally, the ORR responses of the functionalized aerogels are compared (Figure 6d).

Both carbon-doped aerogels exhibit the highest current densities of the series studied, demonstrating that the bonds between iron and carbon play a key role in improving the electrocatalytic response. However, the nature of the doping procedure and, consequently, the amount and type of bonds formed notably influence the reaction. FeA_CA_ does not form a clear plateau but tends to show a direct reaction mechanism, with a slightly defined stage between 0.7 V and 0.45 V, indicating that the material catalyzes the reaction but with low activity for the complete four-electron pathway. Furthermore, the onset potential is low in comparison to FeA and FeA_CVD_. When the iron aerogel is doped with toluene via CVD, the response results in a direct oxygen reduction mechanism (four e^−^ mechanism), with the highest current density obtained (*J* = −4.15 mA·cm^−2^) and a good onset potential. This result is particularly promising compared to other iron-based catalysts in the bibliography. Although they are tested in similar operating conditions, some other electrocatalysts give lower current densities values (i.e., between −0.1 mA·cm^−2^ and −3.0 mA·cm^−2^) [34,35]. On the other hand, some Fe catalysts containing C, with similar current density (−4 mA·cm^−2^) to the FeA presented in this work, require a complex synthesis route [36], hindering their mass production and real application.

**Figure 7:**
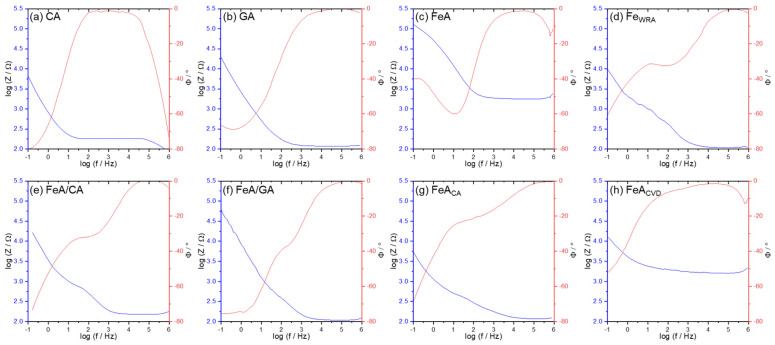
Potentiostatic electrochemical impedance spectroscopy (PEIS) results presented as Bode plots for (**a**) CA, (**b**) GA, (**c**) FeA (**d**) Fe_WRA_ (**e**) FeA/CA, (**f**) FeA/GA, (**g**) FeA_CA_, and (**h**) FeA_CVD_. The blue line represents the impedance variation with frequency on a log-log scale, while the red line represents the phase angle as a function of frequency on a semi-logarithmic scale.

Different chemical processes involved in electrochemical reactions can be studied by electrochemical impedance spectra (Figure 7). It can be clearly observed that, at intermediate frequencies, CA presents higher impedance values, indicating higher intrinsic resistance, in comparison with the GA sample. This can be explained by the presence of graphene in the tridimensional GA structure, which favors electron mobility. The decrease in the impedance signal with the increase in frequency is typical of aerogel materials and their highly porous structure [50,51]. FeA and the functionalized FeA_CVD_ show quite high intrinsic resistance, much higher than the composites or even FeA_CA_, probably due to the open porosity of the former metallic aerogels. The carbon coating observed in FeA_CA_ (Figure 3g) probably favors electric conductivity in this sample.

The phase angle profile of all the samples tends to 0° at high frequencies, indicating resistive behavior [52]. In some cases, a drop in the signal appears at high frequencies, maybe caused by parasitic capacitance or inductive effects in the measurement setup during the data acquisition [53,54]. The presence at mid-frequencies of intermediate peaks in the phase angle for the CA and GA samples indicates that there is no strong capacitive or inductive effect at high frequencies. The smoother transition of the phase angle suggests a more homogenous system where there are no defined transitions between processes like diffusion or charge transfer [55,56].

Although it may appear that the low resistivity of FeA_CA_ favors its electrocatalytic behavior, Figure 5 and Figure 6 clearly show that this is not the case. The FeA_CVD_ sample exhibits much better performance. Impedance measurements provide additional information about the materials, which is useful to have a global idea of the electrocatalyst, but the fundamental characteristic for the applicability of these materials in the ORR is their catalytic activity toward oxygen, and, in this case, both FeA and FeA_CVD_ show better catalytic activity [49].

**Figure 8:**
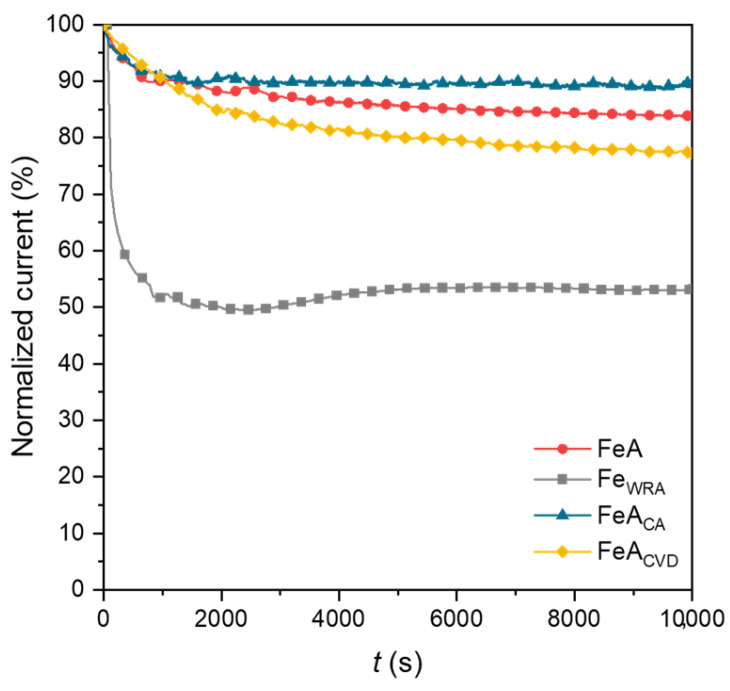
Chronoamperometries of FeA, FeA_WRA_, FeA_CA_, and FeA_CVD_ after 10,000 s, applying a voltage of 0.4 V (RHE).

The stability of the samples was analyzed by chronoamperometry over 10,000 s. The loss of current density was normalized for comparative reasons and is shown in Figure 8. The current density response during the ORR is typically correlated with stability in chronoamperometric measurements. Among the analyzed materials, the reference iron sample (Fe_WRA_) exhibited the lowest stability, showing a pronounced initial drop in current density. Although the current density remains steady after this initial drop, this barely exceeds 50%. This limited stability is attributed to the lack of porosity in the sample, a consequence of the synthesis route, which restricts access to active sites, leading to lower overall activity and stability.

Conversely, the iron aerogel (FeA) demonstrates significantly greater stability, with only a 16% loss in current density, despite not following a direct reaction mechanism and reaching a current density of approximately −2.5 mA·cm^−2^. This improved performance is due to the stability of the aerogel structure and active sites, which remain available and stable throughout the reaction. The two functionalized samples exhibit even higher current density values, indicating that the treatment effectively increases the number of available active sites without compromising structural integrity. Among the doped iron aerogels, FeA_CA_ proves to be the most stable, surpassing the untreated aerogel, with only an 11% loss in current density. In contrast, the CVD-doped sample experiences a greater loss of 23%, likely due to its strong electrochemical response. However, after the initial drop, its current remains stable over time, demonstrating that the electrocatalyst performs effectively following an initial stabilization phase. Given the high activity already shown in Figure 5 and Figure 6, and the maintenance of approximately 80% of the initial current density after 10,000 cycles, this is a very relevant result for this type of material.

## 3. Conclusions

This work highlights the advantage of developing self-supported catalysts, in which a three-dimensional structure greatly facilitates the access and availability of the active centers and favors the stability of the electrocatalyst. Iron aerogel performs better than carbon or graphene aerogels, and even better than their composites with iron aerogel. The right combination of porosity and active sites is essential to achieve an effective and long-lasting electrocatalyst. However, the presence of carbon functionalities seems to play a key role in catalyzing the oxygen reduction reaction. The most relevant role of carbon functionalities is in the ORR mechanism, promoting the direct reaction (four e^−^ mechanism) and avoiding intermediate by-products. The incorporation of carbon to the Fe aerogel by chemical vapor deposition results in an excellent self-supported electrocatalyst. Thus, the 3D structure of the iron aerogel is maintained, the ORR takes place through a direct mechanism, the current density reaches 4.15 mA cm^−2^, and the stability of the electrocatalyst is good after 10,000 cycles, when 80% of the initial current density is maintained. Taking all this into account, the iron aerogel which is C-functionalized by CVD (FeA_CVD_) is presented as a promising material to be used in fuel cells and metal–air batteries. Future research should be focused on the further control and optimization of the C functionalities in order to improve the onset potential.

## 4. Materials and Methods

The iron aerogel (FeA) was prepared by microwave-assisted sol–gel synthesis following the methodology described elsewhere [37]. Briefly, FeCl_2_ (Sigma Aldrich, 98%) was dissolved in distilled water with a concentration of 2 mg mL^−1^. This solution was then mixed with a reducing solution composed of sodium carbonate (Indspec, 99%), glyoxylic acid (Sigma Aldrich, 98%), and deionized water with a mass proportion of 6:1:166. The volumetric ratio between the reducing solution and metal precursor was fixed at 1:4. The mixture was heated at 68 °C for 1 h in a microwave oven, and the solid obtained was washed by centrifugation in a series of 5 cycles of 5 min at 3000 rpm, with deionized water being exchanged in each washing step. To validate the benefits of the metal aerogel’s 3D structure in electrocatalysis, this same procedure was performed without the addition of the reducing agent, so avoiding the promotion of the sol–gel reaction. The solution of FeCl_2_ was heated by microwaves and washed following the same procedure mentioned. This sample without reducing agent was labeled as Fe_WRA_.

The dried FeA was functionalized with carbon following two different strategies. On one hand, FeA was physically mixed with citric acid in an agate mortar in a mass proportion of 1:0.296, to incorporate a 10 wt.% of carbon. Then, the mixture was thermally treated in a nitrogen atmosphere for 2 h at 170 °C with a heating rate and a nitrogen flow of 5 °C min^−1^ and 150 mL min^−1^, respectively, to incorporate the carbon and eliminate the rest of the elements from the citric acid. The second methodology involved the incorporation of carbon by chemical vapor deposition (CVD), using toluene as the carbon precursor. The CVD was carried out for 2 h at 700 °C with a heating rate of 10 °C min^−1^. Initially, the sample was heated at 700 °C under a nitrogen atmosphere (300 mL min^−1^) with a heating rate of 10 °C min^−1^. Then, toluene was introduced with a flow rate of 200 mL min^−1^. This flow was kept for 2 h. Finally, the sample was cooled down naturally under a nitrogen atmosphere (300 mL min^−1^). These samples were labeled as FeA_CA_ and FeA_CVD_, respectively.

To demonstrate the advantages of iron aerogels and its functionalization over the preparation of iron/carbon composites, two additional samples were prepared: (i) a composite involving FeA and the carbon aerogel (FeA/CA) and (ii) a composite involving FeA and the graphene aerogel (FeA/GA). The carbon aerogel (CA) was prepared through the polycondensation reaction between resorcinol (R) (Sumitomo Chemical, Tokyo, Japan) and formaldehyde (F, 37 wt.% formaldehyde solution by Sigma-Aldrich, Burlington, MA, USA), utilizing deionized water as solvent, following the methodology described elsewhere [57,58]. Initially, 50 g of R was dissolved in 100 mL of water, and then 70 mL of F was added under magnetic stirring. Then, this precursor solution was heated in a microwave oven for 5 h at 85 °C to induce the sol–gel reaction: gelation, curing, and drying steps. The graphene aerogel (GA) was synthesized following the same procedure mentioned above but dissolving 10 g of R in 100 mL of a graphene oxide suspension (5 mg mL^−1^, ApplyNano solutions S.L., Alicante, Spain) and adding 15 mL of F, as detailed in previous works [44,59,60]. The pH was adjusted in both cases to a value of 5.0 by adding a 3 M NaOH aqueous solution. After the synthesis, CA and GA were dried by evaporation in an electrical oven at 80 °C overnight and by freeze-drying in a HyperCOOL HC3110 for 48 h, respectively. The dried samples were carbonized in a horizontal electric tubular furnace (Carbolite Type MFT 12/38/400, Eibar, Spain) under a nitrogen flow of 150 mL min^−1^, using a heating rate of 50 °C min^−1^ until a temperature of 1000 °C was achieved, which was kept for 2 h. The composites FeA/CA and FeA/GA were prepared by mechanically mixing in an agate mortar 90 wt.% of FeA with 10 wt.% of CA and GA, respectively.

The textural properties were analyzed by nitrogen adsorption–desorption isotherms measured at −196 °C in a Tristar 3020 (Micromeritics). The samples were outgassed before the analysis at 120 °C and 0.1 mbar overnight in a Micromeritics VacPrep 0.61 station. The specific surface area (S_BET_) was calculated by the BET equation [61], while the volume of micropores was determined by the t-plot method [62]. The morphology of the samples was observed by Scanning Electron Microscopy (SEM) using a Quanta FEG 650 microscope from FEI with an Everhart-Thornley Detector (ETD). An accelerating voltage of 20 kV and a spot size of 3 nm were used in all analyses. The energy-dispersive X-ray analyser Hitachi S-3400 N was employed to determine the chemical composition of the materials and the distribution of the elements through the structure. Thermogravimetric analysis was performed on a TA Instruments TGA Q600. The sample was heated at 5 °C min^−1^ in an airflow of 50 mL min^−1^ from room temperature to 1000 °C.

Electrochemical measurements under hydrodynamic conditions were performed in a biologic multichannel VMP2/Z potentiostat/galvanostat coupled to an RRDE-3A rotating system (©BAS) using a three-electrode cell. The reference, working, and counter electrodes were Ag/AgCl in KCl 3M solution, a glassy carbon electrode with an area of 0.07 cm^2^, and a carbon rod, respectively. The working electrode was modified by depositing a dispersion of 2 mg of the active material, 100 µL of Nafion^®^, and 215 µL of Milli-Q water. This dispersion was sonicated for 30 min before its deposition into the glassy carbon with a mass loading of ca. 0.4 mg cm^−2^. Cyclic voltammetry (CV) and linear sweep voltammetry (LSV) were performed to evaluate the designed materials as electrocatalysts toward the oxygen reduction reaction. CV was performed at a scan rate of 5 mV s^−1^ within a potential range from 0 to 1.1 V (vs. RHE), while the LSV was performed at a scan rate of 5 mV s^−1^ within a potential range from 0 to 1.1 V (vs. RHE) and a rotation speed range of 1600 rpm (adjusted with the RRDE-3A controller). All experiments were performed in a 0.1 M KOH electrolyte saturated with nitrogen and oxygen for 30 min before each experiment. Additionally, the stability of the materials during the ORR was evaluated using chronoamperometry by applying a potential of 0.4 V vs. RHE for 10,000 s, while electrochemical impedance spectroscopy (PEIS) measurements were performed applying a single sinus signal under a fixed potential value of 0 V vs. Eoc and a frequency range between 0.1 Hz and 1 MHz.

## Figures and Tables

**Table 1:** Chemical composition determined by EDX mapping.

Sample	C (wt.%)	O (wt.%)	Fe (wt.%)
FeA/CA	35.0	30.5	34.5
FeA/GA	28.2	35.6	36.2
FeA_CA_	16.7	28.1	55.2
FeA_CVD_	19.6	23.6	56.8

## Data Availability

Data are contained within the article or Supplementary Materials. The original contributions presented in this study are included in the article. Further inquiries can be directed to the corresponding authors.

## Supplementary Materials

The following supporting information can be downloaded at: https://www.mdpi.com/article/10.3390/gels11030154/s1, Table S1: Porous properties obtained from the N_2_ adsorption–desorption isotherms of CA and FeA. Figure S1: SEM images of GA at a magnification of (a) 1000 and (b) 8000.

## Abbreviations

The following abbreviations are used in this manuscript:

CA	Carbon aerogel
CNTs	Carbon nanotubes
CV	Cyclic voltammetry
FeA	Iron aerogel
FeWRA	Iron obtained without reducing agent
FeACA	Iron aerogel doped with C by treatment with citric acid
FeACVD	Iron aerogel doped with C by chemical vapor deposition
GA	Graphene aerogel
LSV	Linear sweep voltammetry
MA	Metal aerogel
NMA	Noble metal aerogel
MNP	Metal nanoparticle
ORR	Oxygen reduction reaction
TMA	Transition metal aerogel
