# Supplementary material for: Revealing the Importance of Iron Aerogel Features as Electrocatalysts for the Oxygen Reduction Reaction
Source: Gels. 2025 Feb 20;11(3):154. doi: 10.3390/gels11030154 (PMC11942411; doi:10.3390/gels11030154)
Supplement: Supplementary file 1 [file gels-11-00154-s001.zip › gels-3481266-supplementary.pdf]

gels

SUPPORTING INFORMATION

Revealing the Importance of Iron Aerogel Features as Electrocatalysts for the Oxygen Reduction Reaction

Judith González-Lavín, Ana Arenillas*, Natalia Rey-Raap*

Instituto de Ciencia y Tecnología del Carbono, INCAR-CSIC, Oviedo, Spain; judith.g.lavin@incar.csic.es
*Correspondence: AA aapuente@incar.csic.es, NRR natalia.rey@incar.csic.es

Table S1 presents quantitative analysis of the information extracted from the N_2 adsorption-desorption isotherms of CA and FeA. GA is not included, as it is just a macroporous material and N_2 adsorption technique is not able to characterize wide macropores.

Table S1. Porous properties obtained from the N_2 adsorption–desorption isotherms of CA and FeA.

Sample	S_{BET} / m^2 g^{-1}	S_{exr} / m^2 g^{-1}	V_{micro} / cm^3 g^{-1}	V_{meso} / cm^3 g^{-1}
CA	565	6	0.22	0.01
FeA	85	81	0.03	0.45

Figure S1 shows SEM images of GA sample in which voids between connected graphene sheets can be clearly observed at a magnification of 1000 (Figure S1a) and 8000 (Figure S1b).

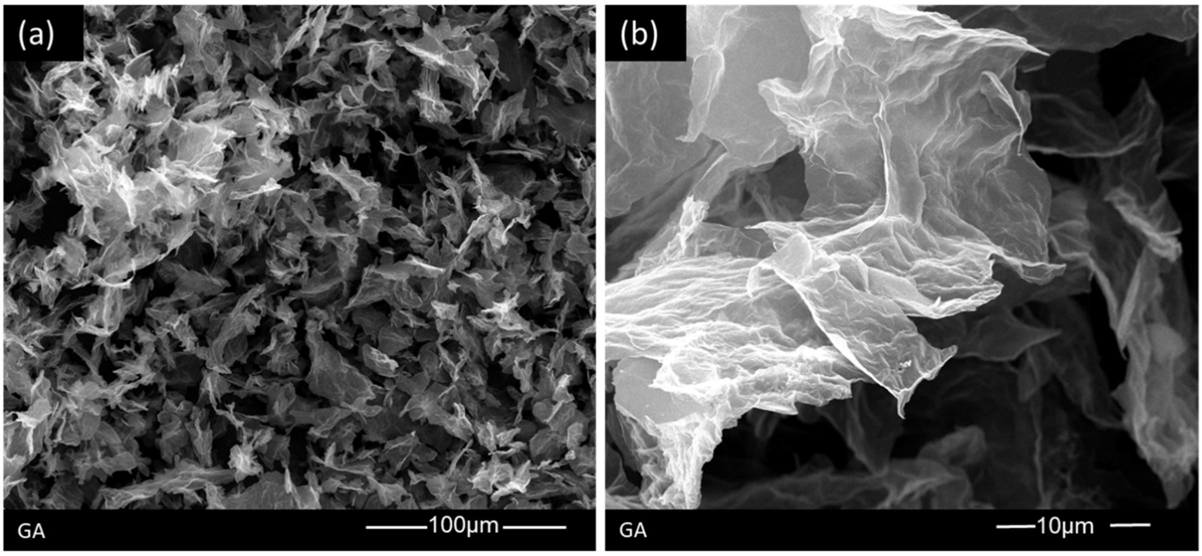

Figure S1. SEM images of GA at a magnification of (a) 1000 and (b) 8000.

 https://doi.org/10.3390/gels11030154
